# Development of a decision support tool to facilitate primary care management of patients with abnormal liver function tests without clinically apparent liver disease [HTA03/38/02]. Abnormal Liver Function Investigations Evaluation (ALFIE)

**DOI:** 10.1186/1472-6963-7-54

**Published:** 2007-04-16

**Authors:** Peter T Donnan, David McLernon, Douglas Steinke, Stephen Ryder, Paul Roderick, Frank M Sullivan, William Rosenberg, John F Dillon

**Affiliations:** 1 Tayside Centre for General Practice, Community Health Sciences, University of Dundee, Dundee, UK; 2 College of Pharmacy, University of Kentucky, Lexington, KY, USA; 3 Directorate of Medicine, Division of Gastroenterology, Queen's Medical Centre, University Hospital NHS Trust, Nottingham, UK; 4 Public Health Sciences and Medical Statistics Group, School of Medicine, University of Southampton, UK; 5 School of Medicine, Division of Infection, Inflammation and Repair, University of Southampton, UK; 6 Division of Pathology and Neurosciences, Ninewells Hospital and Medical School, University of Dundee, Dundee, UK

## Abstract

**Background:**

Liver function tests (LFTs) are routinely performed in primary care, and are often the gateway to further invasive and/or expensive investigations. Little is known of the consequences in people with an initial abnormal liver function (ALF) test in primary care and with no obvious liver disease. Further investigations may be dangerous for the patient and expensive for Health Services.

The aims of this study are to determine the natural history of abnormalities in LFTs before overt liver disease presents in the population and identify those who require minimal further investigations with the potential for reduction in NHS costs.

**Methods/Design:**

A population-based retrospective cohort study will follow up all those who have had an incident liver function test (LFT) in primary care to subsequent liver disease or mortality over a period of 15 years (approx. 2.3 million tests in 99,000 people). The study is set in Primary Care in the region of Tayside, Scotland (pop approx. 429,000) between 1989 and 2003. The target population consists of patients with no recorded clinical signs or symptoms of liver disease and registered with a GP. The health technologies being assessed are LFTs, viral and auto-antibody tests, ultrasound, CT, MRI and liver biopsy.

The study will utilise the Epidemiology of Liver Disease In Tayside (ELDIT) database to determine the outcomes of liver disease. These are based on hospital admission data (Scottish Morbidity Record 1), dispensed medication records, death certificates, and examination of medical records from Tayside hospitals. A sample of patients (n = 150) with recent initial ALF tests or invitation to biopsy will complete questionnaires to obtain quality of life data and anxiety measures. Cost-effectiveness and cost utility Markov model analyses will be performed from health service and patient perspectives using standard NHS costs. The findings will also be used to develop a computerised clinical decision support tool.

**Discussion:**

The results of this study will be widely disseminated to primary care, as well as G.I. hospital specialists through publications and presentations at local and national meetings and the project website. This will facilitate optimal decision-making both for the benefit of the patient and the National Health Service.

## Background

Liver function tests (LFTs) are routinely performed in primary and secondary care, and are often the gateway to further invasive and/or expensive investigations. Little is known of the consequences in people with an initial abnormal liver function (ALF) test [1]. Further investigations such as liver biopsy and endoscopic retrograde cholangiopancreatography may be dangerous for the patient and/or expensive for the National Health Service. Recent guidelines for primary care have been published for evaluation of abnormal liver enzyme results in asymptomatic patients but did not cover other tests nor take account of costs to the patient or the health service [2]. Despite the increasing use of LFTs, patients continue to present with potentially fatal complications of undiagnosed end stage liver disease, which may have been preventable by earlier diagnosis. These include: autoimmune hepatitis which is responsive to steroids, hepatitis C which can be cured in a significant proportion of patients by antiviral drugs, and alcohol misuse [3]. The abnormality of liver function tests may be secondary to serious disease elsewhere requiring treatment, such as malignancy where its early detection may improve the prognosis. Improved patient care demands integration of data from all stages of the patient's illness in order to redesign services appropriately [4,5]. There is a need for quality measures used in the redesign process to be based on routinely collected data rather than instituting specific record searches to address current problems [6].

Most of the published epidemiological studies report only the prevalence of liver disorders rather than addressing the absolute or relative risks of subsequent liver injury following abnormal liver enzyme tests [7-9]. One study examined incidence rates derived from selected hospitalised patients using data from mortality registries [10]. Duh et al, [11] quantified the incidence of liver enzyme abnormalities in the general population but neglected those subjects that subsequently retested normal or did not retest at all, with no long term follow-up to possible liver disease. Although the latter are minor and do not indicate serious disease, they do utilise considerable resources. Recently, a large cohort study in Korea (n = 142 055) reported the association between a key LFT serum aminotransferase (AST and ALT) and mortality from liver disease indicating that even values that were borderline within the normal range were associated with poor outcome [12,13].

Pilot work in Tayside demonstrated that approximately 25% of patients with ALF tests are dead within a year of their first abnormal test result, although this includes those with existing liver disease. A study from Nottingham [14] has reported a similar prevalence of ALF tests and has gone on to investigate the causes, intervening where investigation had not been performed or was inadequate.

### Research objectives

a) To quantify and characterise incident abnormal liver function (ALF) tests in the UK population using data from Tayside and Nottingham. The subjects studied are those with no clinically apparent liver disease with subsequent maximum follow-up over a 15-year period of further investigations, liver disease, non-liver disease and mortality. The study will determine those with no health consequences, those who develop liver disease such as cirrhosis, and its complications, or other liver diseases [see Additional file 1], as well as those who develop serious non-hepatic illness such as cancer. In particular, to determine outcomes at 2, 5 and 10 years and characterise those subjects with:

1) ALF tests that upon investigation are judged as clinically without liver disease;

2) ALF tests that upon investigation are diagnosed with liver disease;

3) ALF tests with no further investigations and apparently normal on follow-up;

4) ALF tests with no further investigations and diagnosed with liver disease on follow-up;

Those who have an initially normal test may also have further tests or no further tests and may or may not develop liver disease. This important group enables estimation of specificity and sensitivity of liver function tests.

b) To devise estimates of the probabilities of disease outcomes following an ALF test with or without further investigations and determine what information would be most useful to clinicians for predicting future patient outcomes and guiding management.

c) To estimate and compare the costs to the National Health Service in terms of LFTs, ultrasound, and other investigative procedures and length of stay in hospital for those with an initial ALF test or normal LFT.

d) To derive decision trees for the various pathways following ALF tests and estimate optimum management of patients in primary care with cost-utility and cost-effectiveness analyses.

## Methods/Design

### Design

A UK population-based observational cohort study will follow up all those who have had an incident abnormal liver function test (ALF) as well as those who were initially normal (to allow calculation of sensitivity, specificity) to subsequent liver disease or mortality. Prior probabilities will be based on pooled measures of prevalence of outcomes in Tayside and Nottingham. Having obtained prior probabilities and probabilities of outcomes from cohort data using logistic or Cox regression modelling, these will be used to create a decision analysis tree, which covers clinically relevant pathways. Finally, the patient survey will provide quality of life measures or utilities to enable cost-utility and cost-effectiveness analyses to be carried out.

### Setting

The study is set within Primary Care in the region of Tayside, Scotland (pop ~. 429,000) between 1989 and 2003. Additionally, primary care data with a median follow-up of 15 months will be provided from Nottingham.

### Target Population

#### Exclusions

**Figure 1:**
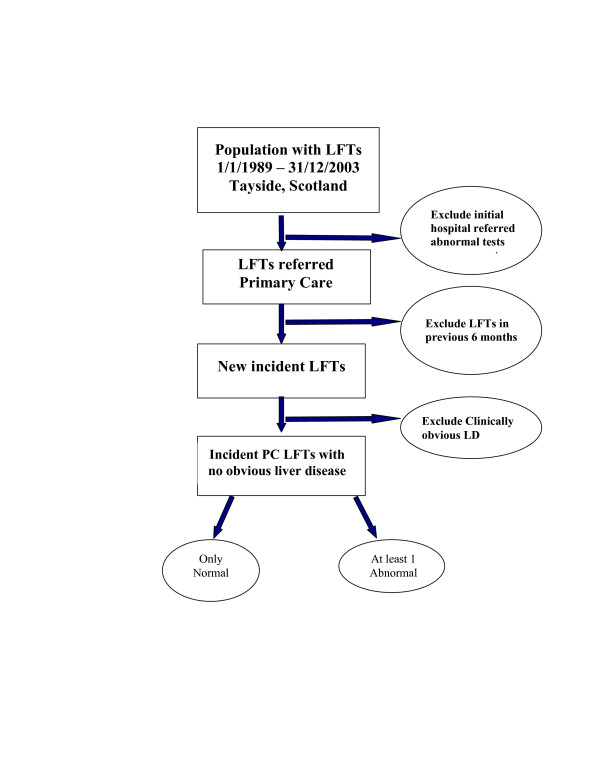
Selection of the study population of Liver function tests with no clinically obvious liver disease.

Patients with no obvious clinical signs and symptoms of liver disease with at least one liver function test and registered with a Tayside GP between 1989 and 2003 will be eligible. A window of 6 months will be used to screen out individuals with already existing liver disease and with previous abnormal LFTs for monitoring purposes. This will ensure only new incident tests in primary care will be included on patients with no clinically obvious liver disease (figure 1).

The following exclusions will ensure that the study population of patients have no clinically obvious liver disease and only includes LFTs referred from primary care:

• Patients under 16

• Patients with liver disease or abnormal LFT in previous 6 months will be excluded

• Based on electronic biochemistry records we will exclude patients whose initial LFTs are hospital referred abnormal LFTs leaving all possible initially abnormal tests requested from primary care

• We will exclude those who have a positive initial bilirubin test (clearly jaundiced at presentation, bilirubin > 35 μmol/l).

• Those with ascites, encephalopathy or variceal bleeding within six weeks of their initial LFTs will be admitted to hospital and can be identified from ELDIT database, SMR1 record as well as spironolactone prescriptions from the Health Informatics Centre database [15].

### Health Technologies being assessed

LFTs antibody tests, ultrasound, CT, MRI and liver biopsy [see Additional file 2]

### Data Sources

#### The Epidemiology of Liver disease in Tayside (ELDIT) database

The ELDIT project created a liver database in Tayside linking administrative clinical data with laboratory data [16]. Briefly, all electronic medical records (including laboratory tests) for Tayside were electronically linked with a unique identifier, the community health index (CHI) [15]. The community health index is used for all health encounters in Tayside for the population registered with a general practice. The following independent data sources were electronically record linked, via the CHI, to maximise the accuracy of diagnosis and disease ascertainment:

• Prescribing database: The Health Informatics Centre (HIC) has person-specific dispensing information for the whole of Tayside [17],

• Hospitalisation, Scottish Morbidity Records (SMR1 – general admissions, SMR4 – alcohol related psychiatric admissions and SMR6 – cancer admissions),

• Death registry from the General Registry Office,

• Carstairs categories for social deprivation based on the decennial census [18],

• Endoscopy,

• Regional biochemistry,

• Pathology,

• Virology, and

• Immunology databases.

Diagnostic algorithms for liver diseases have been created and this database has already been used to assess the epidemiology and economic burden of viral hepatitis [19] and other liver diseases.

The HIC prescription database is complete for all encashed prescriptions for the Tayside population from 1989 to 2003. The hospitalisation records (SMR), and mortality records are 100% complete for all admissions for Tayside residents. There are likely to be gaps in the biochemistry database as in the past, obscure databases were used in some peripheral hospitals and could not be recovered. However, this represents less than 1% of the total data on liver function tests. Given that we will have approximately 2 million tests after exclusions, bias due to missing tests will be minimal.

The ELDIT database as described above will provide robust probabilities of outcomes. Costs of drugs, procedures and hospital admissions will be obtained from standard published values. We are currently updating the ELDIT database to 2003, which is funded by the British Liver Trust.

Prospective questionnaire data from patients undergoing abnormal liver function tests as well as patients undergoing liver biopsy will provide utility-based quality of life measures in order to populate the decision trees. Permission will be sought from the general practitioner as well as the patient. Other utility values will be obtained from the literature and an expert panel of GPs and hepatologists.

Data from the Nottingham study with follow-up of patients in primary care who received an ALF test with a median follow-up of 15 months will also provide information on indication, alcohol, BMI, height and weight.

### Ethics and Data Protection

The proposal has Research Ethics Committee approval as well as the Caldicott Guardians to ensure compliance with the Data Protection Act. All data will be anonymised according to the Standard Operating Procedures (SOPs) of HIC so that the research will be conducted on non-identifiable electronic data.

### Proposed sample size

The annual incidence of ALF tests ranges from 489 to 869 per 100,000 people in the whole Tayside population depending on type of test and year. With a total of approximately 70,000 ALF tests over a 14 year period, of whom approximately 5,500 have liver disease as defined by the ELDIT database, power will be more than adequate (> 90%) to detect relative hazards of the order of 1.2 or greater at the 5% significance level.

### Statistical methods

Descriptive epidemiology for each group as defined by pattern of ALF testing will include analysis of continuous and categorical data on subject characteristics using χ^2 ^tests for categorical variables and by t-tests for continuous variables or non-parametric equivalents.

For the baseline population, liver function tests will be extracted and number and frequency tabulated by year. The 'liver function tests' will be:

Liver function

• Bilirubin

• Albumin

Liver damage

• Alkaline phosphatase (Alk Phos)

• Gamma-glutamyl transferase (GGT)

• Alanine transaminase (ALT)

• Aspartate aminotransferase (AST)(not routinely measured)

Normal and abnormal (and possibly mildly abnormal) categories will be defined for each test using regional laboratory standard cut-offs which vary by age and sex for some tests (table 1). Sensitivity analyses will be performed for some tests (eg ALT where mild abnormality or top quartile in Normal range may be a useful category for prediction.)

**Table 1:** Liver function tests and definitions of abnormal

Liver Function test	Range	Normal (Age & Gender)	Moderately abnormal	Severely abnormal
Bilirubin (μmol/l)	0–1000	<18 M <16 F	18 – 42.5 M 16 – 37.5 F	>42.5 M >37.5 F
Albumin (g/l)	11–60	>35	30–35	<30
Alk Phos (IU/l)	20–2000	120–455 M 16–19	455–1138	>1138
		45 – 195 M 19–26	195–488	>488
		30 – 105 M 26–55	105–263	>263
		45 – 130 M 56–75	130–325	>325
		65 – 150 M 75+	150–375	>375
		120–420 F 16–19	420–1050	>1050
		25 – 90 F 19–26	90–225	>225
		20 – 80 F 26–55	80–200	>200
		40 – 150 F 56–75	150–375	>375
		50 – 190 F 75+	190–475	>475
GGT (IU/l)	5–2000	7 – 42 All 16–24	42–105	>105
		9 – 70 M 24–34	70–175	>175
		11– 75 M 34–44	75–188	>188
		11 – 82 M 44–55	82–205	>205
		11 – 70 M 55+	70–175	>175
		5 – 35 F 24–34	35–88	>88
		5 – 42 F 34–44	42–105	>105
		5 – 65 F 44–55	65–163	>163
		5 – 75 F 55+	75–188	>188
ALT (IU/l)	12–9999 depending on age and sex	14 – 40 M 16–18	40–100	>100
		15 – 55 M 18–55	55–138	>138
		12 – 35 F 16–18	35–88	>88
		12 – 40 F 18–55	40–100	>100
		13 – 43 All 55–75	43–108	>108
		6 – 30 All 75+	30–75	>75
AST (IU/l)		3 – 30 M 17–65	30 – 75	>75
		10 – 45 F 16–75	45 – 113	>113
		10 – 30 75+	30 – 75	>75

Categories of patterns of tests will be defined. For example the following may be possible:

1. Raised ALT + normal Alk Phos + normal GGT (suggesting hepatitis)

2. Raised Alk Phos +/- raised GGT + normal ALT (suggesting biliary cirrhosis)

3. Any one abnormal

4. Any 2 or more abnormal and/or explore patterns.

Patterns of repeat tests will also be explored. The main outcomes and covariates will be tabulated by initial and repeat LFT patterns (Rates presented as events per thousand person years).

Sensitivity, specificity, positive predictive value and likelihood ratios will be calculated for LFT patterns compared with actual outcome. Kaplan-Meier plots of time to outcomes will be plotted for patterns of tests and individual tests above.

### Derivation of probabilities

Fixed 1, 2, 5 and 10-year probabilities of outcomes will be estimated from logistic regression analyses. Probabilities of outcome of liver disease or all cause mortality will be calculated using the Cox proportional hazards regression model [20] adjusting for confounders (such as age, sex, co-morbidities, social deprivation), possibly incorporating time dependent covariates and between subject heterogeneity using frailty terms [21]. As deriving probabilities from the Cox model is not trivial, involving estimation of the baseline hazard, an alternative is the Weibull parametric regression model which easily allows estimation of probability of outcome over any time period. The Weibull accelerated failure time model has been used to derive the Framingham coronary heart disease (CHD) risk equation [22] and a CHD risk score for type 2 diabetes from Tayside data [23]. This gives greater flexibility in modelling over different time periods.

The main outcomes will be all-cause mortality (Yes/No); Liver disease mortality (Yes/No); Liver disease such as non-alcoholic fatty liver disease (NAFLD), alcohol related liver disease, viral hepatitis and primary biliary cirrhosis (PBC) (Yes/No); Any hospitalization; Cirrhosis/portal Hypertension.

The above models will also allow derivation of risks of outcomes stratified by factors entered in the regression model. For example, the risk of liver disease is clearly greater in patients with known alcohol abuse compared to those without alcohol abuse. The following factors will be entered in the regression models to assess their effect on risk and estimate factor-specific risks:

1) Further tests such as repeated LFTs, liver biopsy, ultrasound, virology, immunology and ferritin as well as time between tests. Other baseline stratification factors may be:

2) Age – derived from the first 6 digits of patient identifier, the community health index (may be categorized as < 40 and 40+ depending on age distribution);

3) Gender – derived from the 9^th ^digit of the patient identifier, the community health index;

4) Pregnancy – from hospitalisation records, SMR2. This will, of course, miss home births;

5) Opioid abuse – A proxy measure will be obtained using methadone prescribing from the HIC prescription database

6) Alcohol abuse – We will use hospitalisation records from SMR1 and SMR6 which includes ICD10 codes F10, X65 and T51. Y90 and Y91 may be used as supplementary information. This will represent the more extreme end of alcohol abuse which is a weakness in missing others with mild or moderate alcohol abuse. On the other hand, this is also a strength in giving a clear definition and measures of the drivers of costs to the health service. The alternative of general practice notes would be prohibitively expensive and prone to classification error.

7) Social deprivation – The Carstairs social deprivation score assigned to postcodes for all residents of Tayside, is derived from the decennial census, incorporating the variables, housing density, car ownership, social class of the head of household and male unemployment [18].

Although social deprivation is a marker for cigarette smoking and co-morbidity, we will also be able to assess the affect of individual co-morbidities on risk.

8) Diabetes will be defined from the Diabetes Audit and Research in Tayside, Scotland (DARTS) database which is 97% sensitive for ascertainment of diabetes in the population [23].

9) The Hearts database (sensitivity 95%) will identify those who have definite CHD (myocardial infarction or demonstrated coronary artery disease) in the Tayside population [24].

Other major co-morbidities [see Additional file 3] such as

10) respiratory disease

11) cerebrovascular disease,

12) renal disease,

13) ischaemic heart disease,

14) cancers other than liver cancer

will be defined by the Scottish Morbidity Record which contains details of all hospital admissions, including ICD9, ICD10 codes, for all Tayside residents, which is held in HIC.

HIC also contains the database of all encashed prescriptions in Tayside. This resource can also be used to create co-morbidity variables such as

15) Psychotropic drugs: monoamine oxidase inhibitors, Phenothiazines

16) Analgesics: NSAIDS, paracetamol, aspirin

17) Antibiotics

18) Lipid lowering agents such as statins and fibrates

19) sodium valproate

20) oestrogens (oral contraceptives, HRT)

Importantly, this resource will allow us to identify receipt of prescribed hepatotoxic drugs at the time of any ALF test.

Costs which are invariably positively skewed will be summarised and compared between cohorts using bootstrapping procedures for means and confidence intervals [26,27]. Statistical analysis will be performed on SAS version 8.0 (SAS Institute, Cary, NC)

### Decision analyses

Decision analysis is the formal process whereby the probabilities of outcome events, such as liver disease, are combined with patients' preferences or values. The approach is most useful in informing clinical decisions where the optimal pathway is not immediately apparent, and for making clinical reasoning explicit [28]. Hence, these analyses will inform the management of patients with an abnormal liver function test, but who are otherwise well. The probabilities of outcomes are generally derived from regression analysis of cohort studies of large populations or from previous published results. The analysis of the Tayside population will provide robust estimates of these probabilities stratified by important confounders. For example, the measurement of alkaline phosphatase is known to be dependent on age, gender, and blood type and so the probability estimate needs to be adjusted appropriately. The Weibull regression analyses described above can be enhanced using a Bayesian approach whereby prior probabilities (prevalence or previous published results) can be updated by new data, utilizing the software WinBUGS.

Utilities can be extracted from previously published work but it is likely this form of research is sparse in liver disease. A utility of 1 is taken to represent optimal health, while 0 represents death. Utilities are combined with length of time in a condition or state to give quality adjusted life years (QALYs), where QALY = QOL multiplied by length of time in the state. For example, a commonly quoted utility for stroke is 0.75, meaning that 4 years suffering from stroke has a QALY = 3 years. Alternatives to published results include panels of liver disease experts constructing values that appear to have face value. Ideally, utilities can be obtained directly from patients using QOL utility measures such as EQ-5D. This instrument is easily used in questionnaires and has 5 dimensions, each with three levels generating 243 theoretically possible health states covering mobility, self-care, usual activities, pain/discomfort, anxiety/depression. The prospective questionnaire for patients undergoing a liver function test and patients undergoing liver biopsy will provide utility data essential for the cost-utility analysis in which the decision analysis aims to maximize expected utility. This questionnaire survey will focus on the more serious investigations such as liver biopsy, as the utility of a single liver function test such as bilirubin in an otherwise healthy patient is likely to be close to 1. A literature search will enable us to find and possibly pool estimates of liver diseases such as hepatitis C and various stages of cirrhosis.

**Figure 2:**
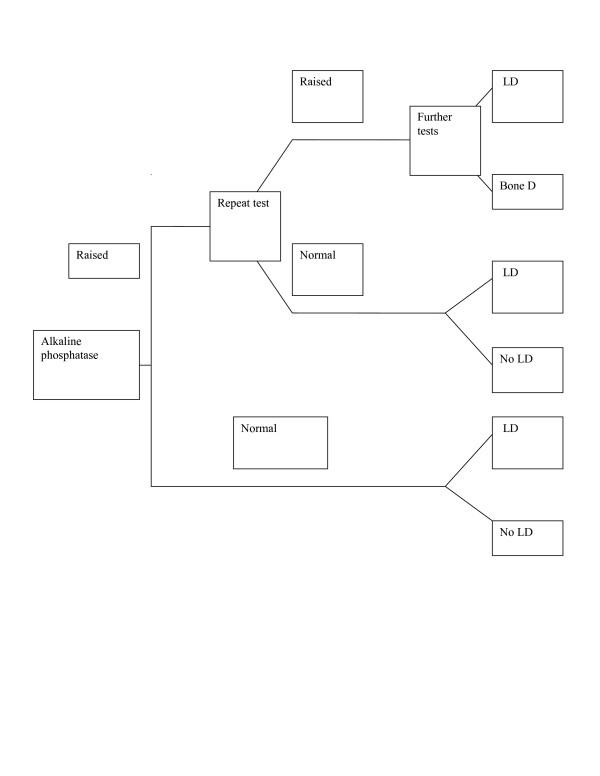
Example of possible simplified decision analysis tree following alkaline phosphatase test (LD – liver disease, based on Pratt and Kaplan, 2000).

Decision analyses also allow other measures such as costs to be incorporated and the analysis aims to minimize costs or maximize cost-effectiveness from a health service perspective. An example of how a decision tree for alkaline phosphatase might look is given in figure 2. This has been simplified for presentation, and is based on Pratt and Kaplan [2]

One limitation of decision analysis is that the time horizon is generally short and costs, utilities and probabilities may vary over time as well as by type of liver disease. However, the approach of Markov modeling allows extension of time indefinitely and hence is useful for modelling cost and utilities until death of all members of the cohort [29]. The Markov process simulates movement of an individual or cohort through various states, each of which is associated with a probability, utility and cost. As some liver disease may not be apparent in a short period of time Markov modelling will be utilized in modelling cost-effectiveness and cost-utility analysis over longer time periods.

As the probabilities, cost and utilities are fixed estimated values in decision analysis, it is more realistic to allow for error of these measures using Bayesian methods in probabilistic sensitivity analyses. Decision analyses will utilize the software Data 4 (TreeAge).

### Proposed outcome measures

The study will use the epidemiology of liver disease database (ELDIT) in Tayside, and the Nottingham cohort to determine the outcome of liver disease (as listed in appendix 2). In Tayside, outcomes of the extent of liver disease and co-morbidities of subjects with no retest or subsequent normal liver function tests will be determined by hospital admission data (Scottish Morbidity Record 1), dispensed medication records, death certificates, and examination of medical records from Tayside hospitals. Those with normal tests are less likely to be investigated and hence classified with liver disease on death certificates. Simulation of likely misclassification rates will demonstrate whether this potential bias has a large influence on results. Sensitivity, specificity, positive and negative likelihood ratio and yield (1/PPV) will be calculated for each pathway. All liver function tests (LFTs) will be obtained from the ELDIT and biochemistry databases and standard costs applied. All procedures and hospitalisations of all subjects will be obtained from the Tayside portion of the SMR1 record and the cost per day by speciality and hospital will be applied from published NHS costs. Future costs will be discounted at 6% per year, where necessary.

Cost-effectiveness and cost utility analyses will be performed from health service and patient perspectives. Cost effectiveness will seek to minimise the cost of investigations per case of liver disease detected, while cost-utility analyses will seek to maximize expected utility. In the Markov models effectiveness is measured as life expectancy by risk factor strata, adjusted using utilities to give QALYs.

The findings will also be used to develop a computerised clinical decision support tool. This will be evaluated and validated in a further study as the subject of a separate application as part of a program of research in Dundee's Health Informatics Centre (HIC), where funded development of decision support tools in cardiovascular risk, caesarean delivery, breast and colorectal cancer are already underway. The results of this study will be widely disseminated to primary care, as well as G.I. hospital specialists through publications and presentations at local and national meetings and the project website This will facilitate optimal decision-making both for the benefit of the patient and the National Health Service.

## Discussion

### Consumers

The Health Informatics Centre has developed a User Participation Group for input from the consumer for research ideas, development of research tools, and dissemination. The views of this group will be sought in developing the study materials in relation to the patient survey and in dissemination of the results.

### Impact on and benefit to the NHS

Abnormal liver enzymes may indicate liver injury which is asymptomatic in the early stages and subsequent testing may diagnose symptomatic liver disease. The probability of disease is unknown. The sequence of subsequent tests is at the discretion of the practitioner. The care pathway will give clinical sequencing for subsequent testing and follow-up, thus maximising the probability of diagnosis and eliminating unnecessary expense to the NHS and unwanted patient trauma. The decision support system developed could be used in conjunction with the electronic results communications system within Tayside, which could be subsequently rolled out across the NHS. Tayside is a lead site for Scotland's Electronic Clinical Communications Initiative that has the philosophy of a single clinical web-based repository linked to the locally developed Area Community Health Index. The Tayside Core Network connects every GP practice and hospital within Tayside with a single access to NHSNet and presently has 88 sites with over 4000 PCs connected. This proposal will be developed, within the IT framework established by the Board and the Trusts to ensure that the decision support system, this project develops can be adequately supported by them and demonstrate the practicality for the wider NHS.

In summary, this study will determine the natural history of abnormalities in LFTs before overt liver disease presents in the population and will also provide identification of those who require minimal further investigations with the potential for reduction in NHS costs.

## Abbreviations

LFT – Liver function tests

ALF – Abnormal liver function

ELDIT – Epidemiology of Liver Disease in Tayside

HIC – Health Informatics Centre

SMR – Scottish Morbidity Record

CHI – Community Health Index

QALY – Quality Adjusted Life Year

ALT – Alanine transaminase

GGT – Gamma-glutamyl transferase

AST – Aspartate aminotransferase

## Competing interests

The author(s) declare that they have no competing interests.

## Authors' contributions

All authors contributed to the writing of the study protocol and approved the final version. The study was initiated by PTD, JD, FS and DMcL. The ELDIT database was developed by JD, DS, DMcL and PTD. SR, WR, PR provided leading UK liver disease expertise.

## Pre-publication history

The pre-publication history for this paper can be accessed here:



## Supplementary Material

Additional File 1 Appendix 1. Indications for liver function tests with no obvious liver disease, and consequent investigations Click here for file

Additional File 2 Appendix 2. Possible Outcomes following abnormal liver function tests Click here for file

Additional File 3 Appendix 3. ICD9/ICD10 codes for liver disease, comorbidities and other outcomes Click here for file
