# Supplementary material for: Development of a decision support tool to facilitate primary care management of patients with abnormal liver function tests without clinically apparent liver disease [HTA03/38/02]. Abnormal Liver Function Investigations Evaluation (ALFIE)
Source: BMC Health Serv Res. 2007 Apr 16;7:54. doi: 10.1186/1472-6963-7-54 (PMC1868021; doi:10.1186/1472-6963-7-54)
Supplement: Additional File 1 — Appendix 1. Indications for liver function tests with no obvious liver disease, and consequent investigations [file 1472-6963-7-54-S1.doc]

Appendix 1 Possible Outcomes following abnormal liver function tests

|  |
| --- |
| No retest |
| LFTs normalize without intervention |
| LFTs normalize after alcohol and / or weight reduction advice |
|  |
| Acute hepatitis A-E, EBV, CMV, toxaplasmosis |
| Chronic hepatitis B, C-carrier/disease |
|  |
| Gallstones |
| Shock liver |
| Post-operative cholestasis |
| Acute fatty liver of pregnancy |
| Cholestasis of pregnancy |
|  |
| ALF test due to adverse drug reaction |
| Non alcoholic fatty liver disease (NAFLD) |
| Non alcoholic steato-hepatitis (NASH) |
|  |
| Alcoholic liver disease/fatty liver |
| Alcoholic Hepatitis |
| Alcoholic Cirrhosis |
|  |
| Ideopathic cirrhosis |
| Primary Biliary Cirrhosis (PBC) |
| Auto-immune hepatitis |
| Haemochromatosis |
| Alpha-1-anti-trypsin |
|  |
| Metastatic cancer |
| Liver cancer |
| Pancreatic cancer |
| Para-neoplasmic syndrome |
| Congestive heart failure |
| Systemic inflammatory conditions (arthritis, vasculitis, etc.) |
|  |
