# Supplementary material for: Development of a decision support tool to facilitate primary care management of patients with abnormal liver function tests without clinically apparent liver disease [HTA03/38/02]. Abnormal Liver Function Investigations Evaluation (ALFIE)
Source: BMC Health Serv Res. 2007 Apr 16;7:54. doi: 10.1186/1472-6963-7-54 (PMC1868021; doi:10.1186/1472-6963-7-54)
Supplement: Additional File 2 — Appendix 2. Possible Outcomes following abnormal liver function tests [file 1472-6963-7-54-S2.doc]

Appendix 2 Indications for liver function tests with no obvious liver disease, and consequent investigations

| Indications | Investigations |
| --- | --- |
|  |  |
| None | Liver function test |
| Other monitoring | Bilirubin |
| (e.g. cholesterol) | Albumin |
| Health check | Liver enzyme tests |
| Tired all the time | Alanine transaminase |
| Nausea | Aspartate aminotransferase |
| Alcohol abuse | Alkaline phosphatase |
| Unwell | γ-glutamyltransferase |
| Health check |  |
|  | Abdominal ultrasound |
|  |  |
|  | Haematology and clotting |
|  |  |
|  | Immunology |
|  | ASMA |
|  | ANF |
|  | AMA |
|  | ANCA |
|  |  |
|  | Virology |
|  | HBV antibodies and DNA |
|  | HCV antibody and RNA |
|  | Antibodies to other viruses |
|  |  |
|  | Biochemistry |
|  | Ferritin |
|  | Immunoglobulins |
|  | Alpha-1-antitrypsin |
|  | Ceaurloplasmin |
|  |  |
|  | Genetics |
|  | Haemochromatosis genotype |
|  | Gilberts genotype |
|  |  |
|  | Radiology |
|  | Computerised Tomography Scan (CT scan) |
|  | Magnetic Resonance Imaging (MRI) |
|  |  |
|  | Endoscopy |
|  |  |
|  | Liver biopsy |
