# Supplementary material for: Development of a decision support tool to facilitate primary care management of patients with abnormal liver function tests without clinically apparent liver disease [HTA03/38/02]. Abnormal Liver Function Investigations Evaluation (ALFIE)
Source: BMC Health Serv Res. 2007 Apr 16;7:54. doi: 10.1186/1472-6963-7-54 (PMC1868021; doi:10.1186/1472-6963-7-54)
Supplement: Additional File 3 — Appendix 3. ICD9/ICD10 codes for liver disease, comorbidities and other outcomes [file 1472-6963-7-54-S3.doc]

Appendix 3 ICD9 / ICD10 codes for liver disease, comorbidities and other outcomes

| **Disease** | **ICD 9** | **ICD 10** | **Other source/notes** |
| --- | --- | --- | --- |
| **Liver disease diagnosis** |  |  |  |
| Hepatitis B |  |  | Virology |
| Hepatitis C |  |  | Virology |
| Autoimmune hepatitis | 571.4 | K73.0, K73.2, K73.8, K73.9 | Pathology, immunology (+ve ASM) |
| Cirrhosis | 571.2, 571.5, 571.6 | K70.3, K74.3-K74.6, K76.1 | Pathology |
| PBC |  |  | Pathology, immunology (+ve AMA), biochemistry (+ve GGT, +ve Alk Phos) |
| Alcoholic cirrhosis | As cirrhosis + 291, 303, 305.0 | As cirrhosis + F10 | SMR1/SMR4 ICD codes for alcohol |
| Alcoholic hepatitis | 571.1 | K70.1 | Pathology |
| Alcoholic related liver disease | Any liver disease codes+alcohol codes | Any liver disease codes+alcohol codes | Pathology |
| Fatty liver disease | 571.0, 571.8 | K70.0, K76.0 | Pathology |
| Hepatocellular carcinoma | 155.0, 155.2 | C22.0, C22.2-C22.9 | Pathology (ICD from SMR1/SMR6) |
| Wilson’s disease | 275.1 | E83.0 |  |
| Haemochromatosis | 275.0, 285.0 | D64.2, E83.1 | Pathology |
| Alpha 1-antitrypsin |  |  | Biochemistry (+ve Alpha 1-antitrypsin) |
| **Complications** |  |  |  |
| Oesophageal Varices | 456.1, 456.2 | I85.9, I98.2A | Endoscopy records |
| - bleeding | 456.0 | I85.0 |  |
| Gastric varices |  | I86.4 |  |
| Ascites | 789.5 | R18X |  |
| Encephalopathy | 572.2 | K72.9 |  |
| Portal hypertension | Any complication, 572.3 | Any complication, K76.6 |  |
| **Diseases of gallbladder & biliary tract** |  |  |  |
| Cholelithiasis | 574 | K80 |  |
| Other disorders of gallbladder | 575 | K81-K82 |  |
| Other disorders of biliary tract | 576 | K83 |  |
| Cholangiocarcinoma | 155.1, 156-157, 230.8 (in-situ) | C22.1, C23-C25, D01.5 (in-situ) | ICD from SMR1/SMR6 |
| **Comorbidities** |  |  |  |
| Ischaemic heart disease | 410-414 | I20-I25 |  |
| Other cancers | 140-208, 230-234 (exclude cholangiocarcinoma and hepatocellular codes) | C00-D09 (exclude cholangiocarcinoma and hepatocellular codes) |  |
| Diabetes | 250 | E10-E14 |  |
| Respiratory | 466, 480-496 | J10-J18, J20, J40X-J47X, J66-J67 |  |
| Renal | 584-586 | N17-N19X, I12.0 |  |
| Stroke | 430-438 | I60- |  |
| **Other liver conditions n.b. only on death cert.** |  |  |  |
| Acute/subacute necrosis | 570.9 |  |  |
| Alcoholic liver damage unspec | 571.3 | K70.9 |  |
| Chronic hepatitis | 571.4 |  |  |
| Non-alcoholic CLD unspec | 571.9 |  |  |
| Abscess of liver | 572.0 | K75.0 |  |
| Hepatorenal syndrome | 572.4 | K76.7 |  |
| Other sequelae of CLD | 572.8 |  |  |
| Other liver disorder eg hepatoptosis | 573.8 | K76.8 |  |
| Unspec liver disorder | 573.9 | K76.9 |  |
| Alcoholic hepatic failure |  | K70.4 |  |
| Acute/subacute hepatic failure |  | K72.0 |  |
| Chronic hepatic failure |  | K72.1 |  |
| Occlusion of vena cava | 453.2 | I82.2 |  |
| Portal vein thrombosis | 452 | I81 |  |
| Hepatitis B | 070 | B16, B18.0, B18.1 |  |
| Hepatitis C | 070 | B17.1, B18.2 |  |
| Other viral hepatitis | 070 | B17-19 |  |
